# Supplementary figures and images for: Structural and functional insights into a novel two-component endolysin encoded by a single gene in Enterococcus faecalis phage
Source: PLoS Pathog. 2020 Mar 16;16(3):e1008394. doi: 10.1371/journal.ppat.1008394 (PMC7098653; doi:10.1371/journal.ppat.1008394)

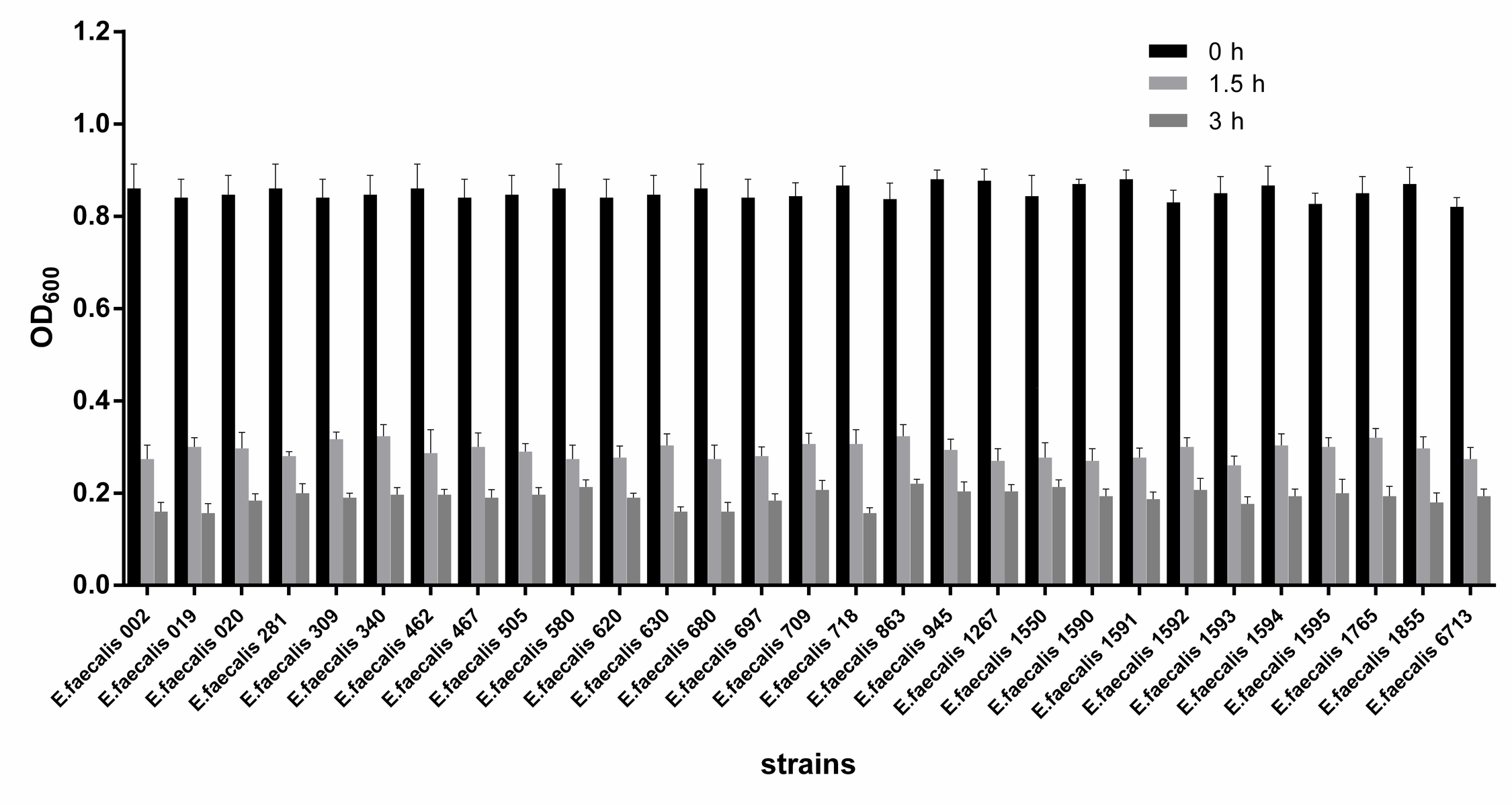

Supplement: S1 Fig — A decrease in OD600 was used to evaluate the lytic activity of LysIME-EF1. WT LysIME-EF1 was used to lyse against E. faecalis. All assays were performed in triplicate, and the data are expressed as means ± SD (n = 3). (TIF) [file ppat.1008394.s001.tif]

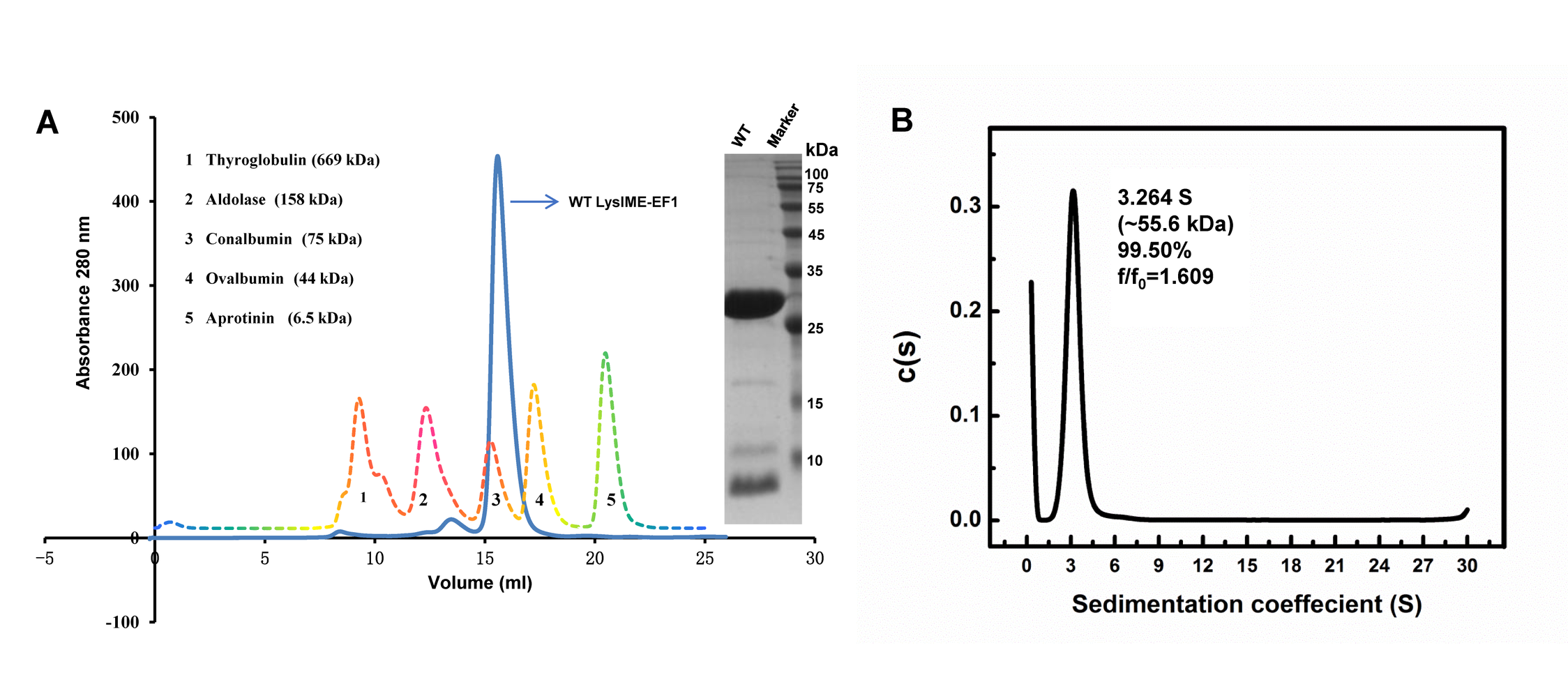

Supplement: S2 Fig — (A) Size-exclusion chromatography (GE Healthcare, Superdex 200 increase, buffer: 20 mM Tris-HCl, pH 8.0, 150 mM NaCl) of WT LysIME-EF1. The CBD fragment of LysIME-EF1 was co-eluted with the full-length LysIME-EF1 at 15.5 mL, standard proteins containing Thyroglobulin (669 kDa), Aldolase (158 kDa), Conalbumin (75 kDa), Ovalbumin (44 kDa), Aprotinin (6.5 kDa) were performed in the same buffer. (B) Analytical ultracentrifugation (AUC) of the exact molecular mass of WT LysIME-EF1, which yielded a sedimentation coefficient of 3.264 S with a molecular mass of 55.6 kDa. The c(s) represents continuous (sedimentation coefficient distribution) analysis model. (TIF) [file ppat.1008394.s002.tif]

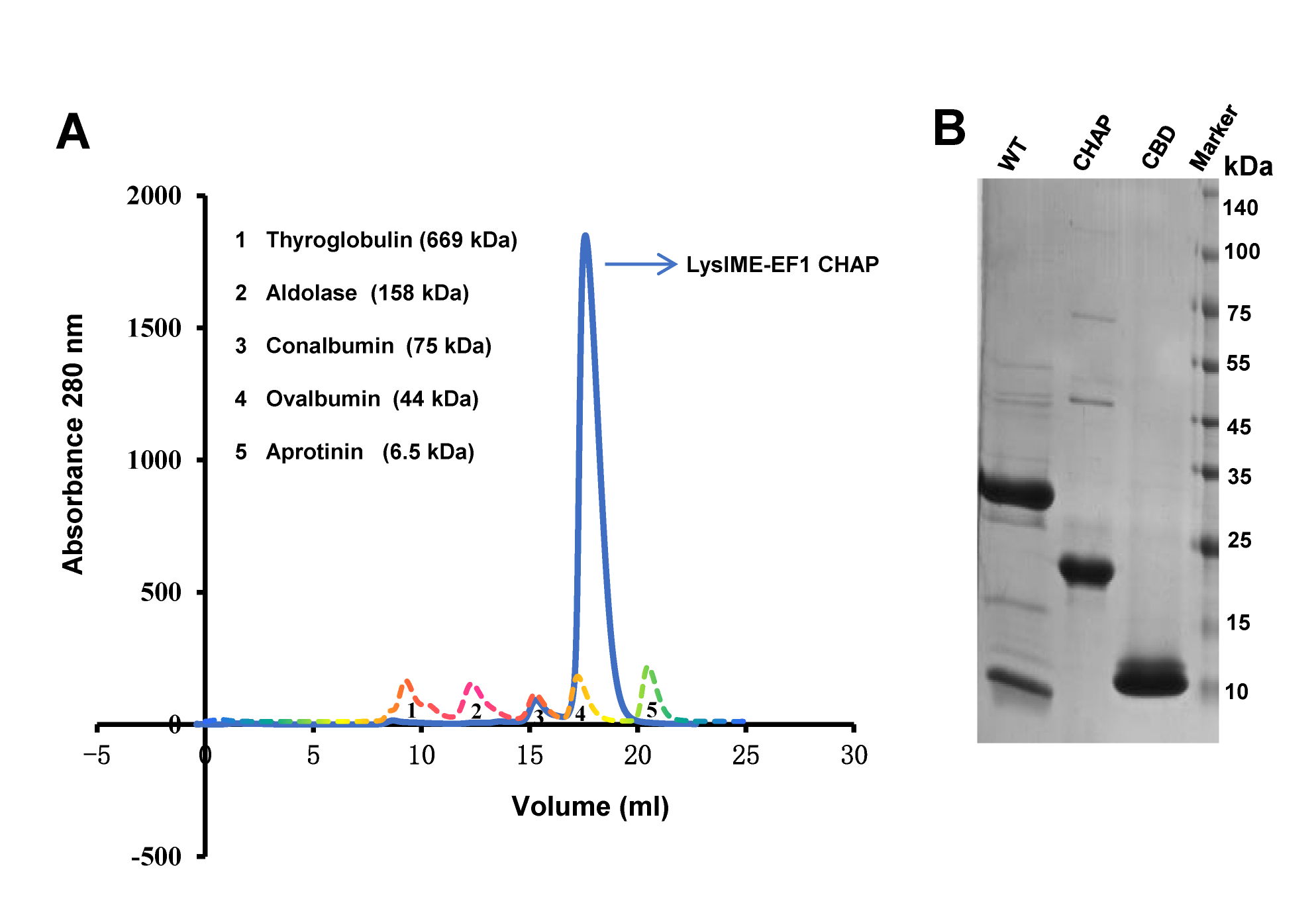

Supplement: S3 Fig — (A) Size-exclusion chromatography (GE Healthcare, Superdex 200 increase, buffer: 20 mM Tris-HCl, pH 8.0, 150 mM NaCl) of LysIME-EF1 CHAP, standard proteins containing Thyroglobulin (669 kDa), Aldolase (158 kDa), Conalbumin (75 kDa), Ovalbumin (44 kDa), Aprotinin (6.5 kDa) were performed in the same buffer. (B) The SDS-PAGE analysis of LysIME-EF1 CHAP and CBD. (TIF) [file ppat.1008394.s003.tif]

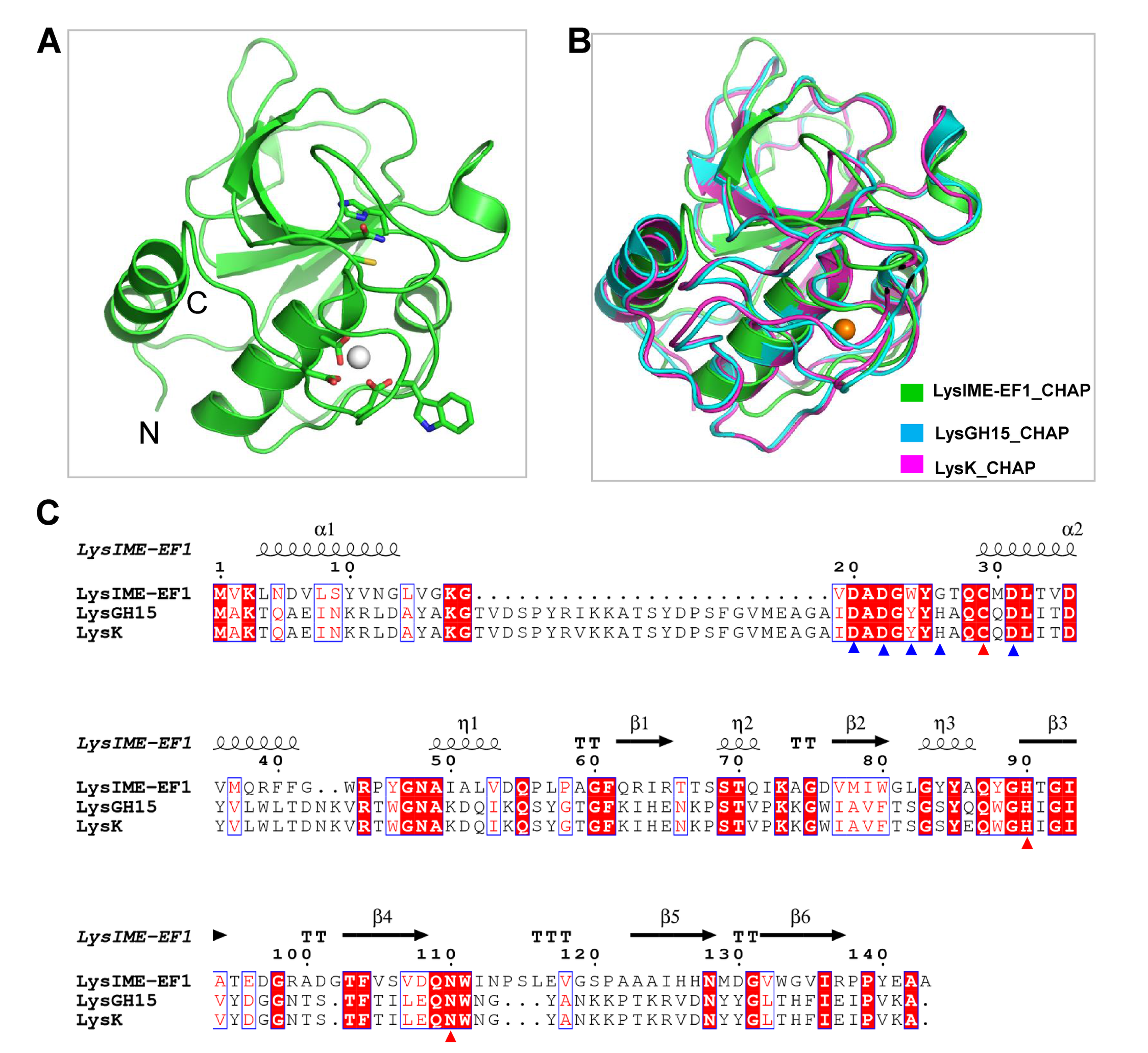

Supplement: S4 Fig — (A) The structure of the CHAP domain of LysIME-EF1. (B) The superposition of the CHAP domains of LysIME-EF1, LysK and LysGH15. (C) Structure-based sequence alignment of LysIME-EF1, LysGH15 and LysK CHAP domain. Strictly conserved residues are boxed in white on a red background and highly conserved residues are boxed in red on a white background. At the top of the sequences, the secondary structure elements of LysIME-EF1 CHAP are shown, and every 10 residues are indicated with a dot (.) shown above the sequences. Sequence alignment was generated by ClustalW (https://www.genome.jp/tools-bin/clustalw) and the figure was generated by ESpript 3 (http://espript.ibcp.fr/ESPript/ESPript/). The blue triangles indicate the residues involved in Ca2+ ion binding and the red triangles indicate the catalytic triad residues on CHAP domain. (TIF) [file ppat.1008394.s004.tif]

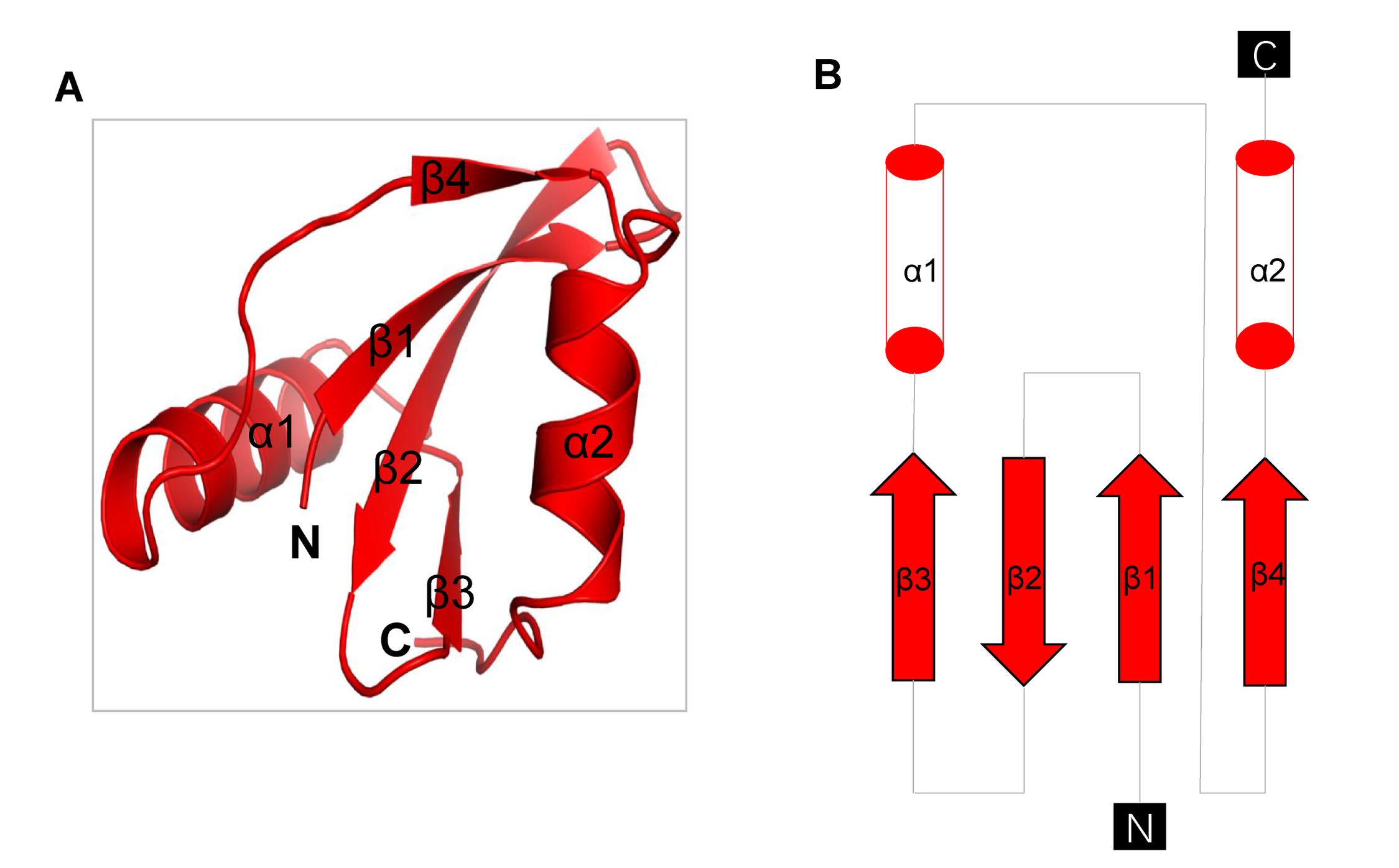

Supplement: S5 Fig — (A) Overview of LysIME-EF1 CBD monomer with labeled secondary elements. The LysIME-EF1 CBD monomer was shown in red color. (B) Topological diagram of LysIME-EF1 CBD with β-strands drawn in black arrows and the α-helices shown as cylinders. (TIF) [file ppat.1008394.s005.tif]

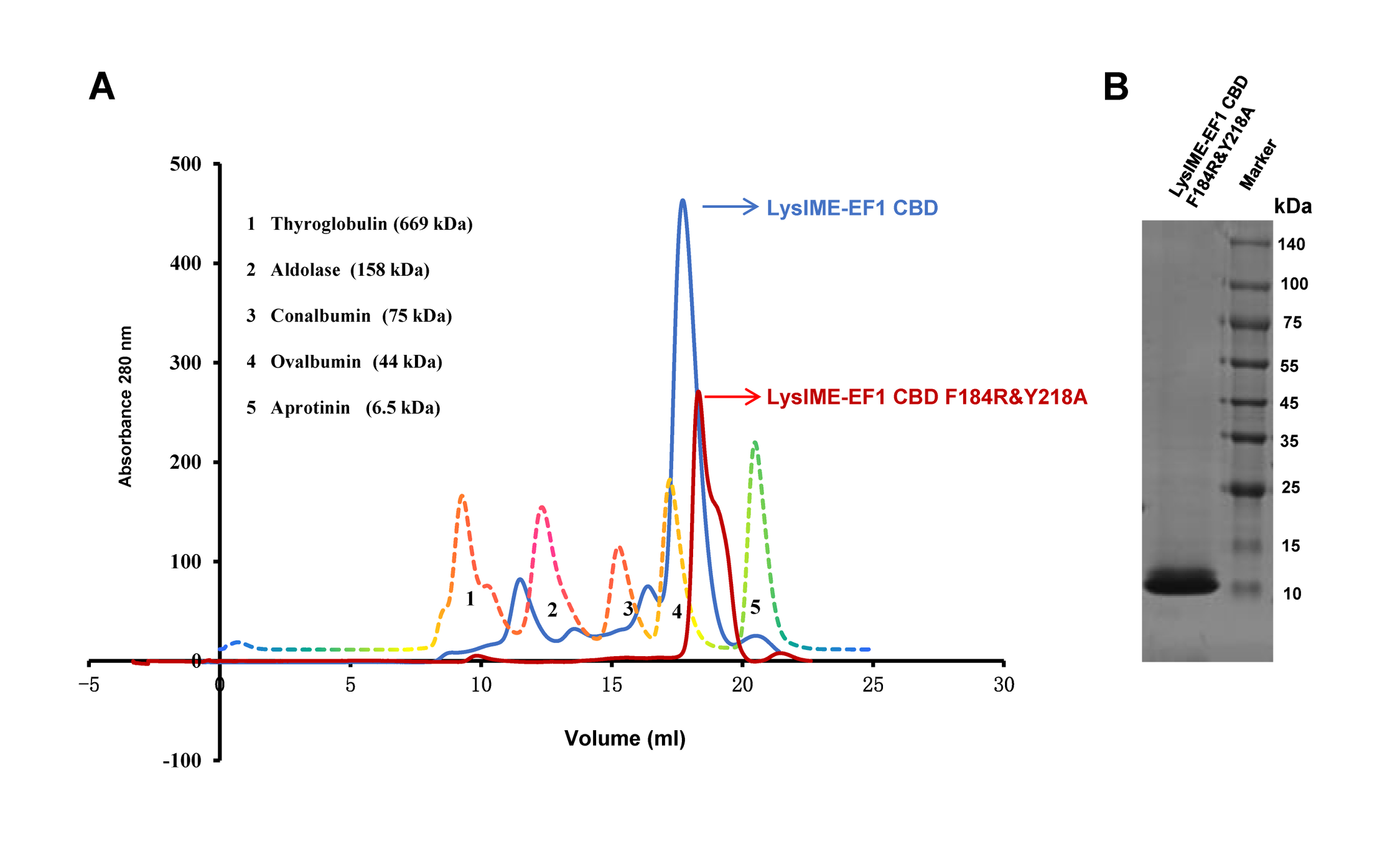

Supplement: S6 Fig — (A) Size-exclusion chromatography (GE Healthcare, Superdex 200 increase, buffer: 20 mM Tris-HCl, pH 8.0, 150 mM NaCl) of LysIME-EF1 CBD and the mutant LysIME-EF1 CBD F184R/Y218A. The elution peaks for the two proteins were 17.7 ml and 18.7 ml, respectively. (B) The SDS-PAGE analysis of LysIME-EF1 CBD F184R&Y218A protein. (TIF) [file ppat.1008394.s006.tif]

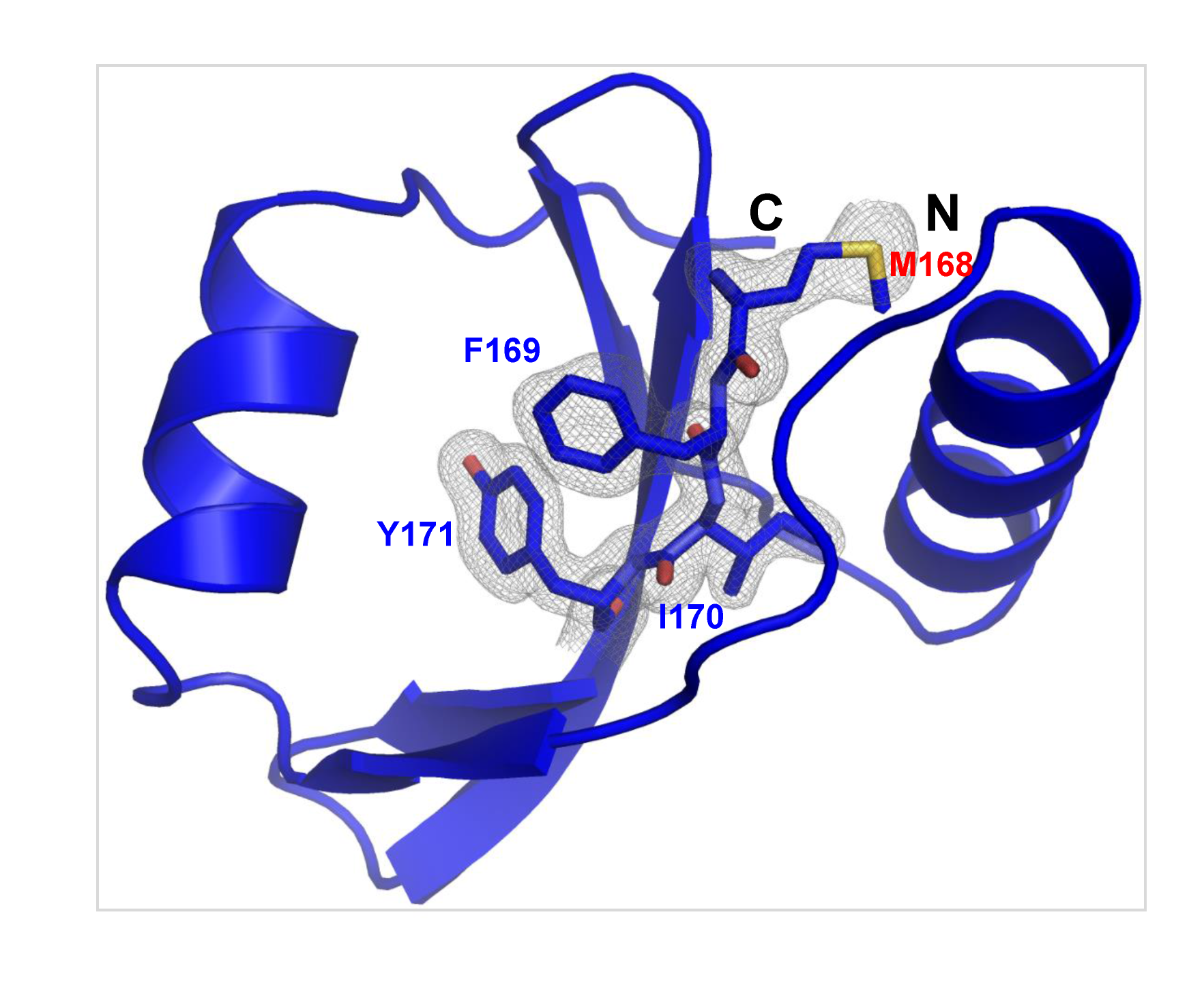

Supplement: S7 Fig — A blue colored monomer extracted from the structure of Fig 4A. The first four residues of LysIME-EF1 CBD including M168F169I170Y171 are shown in sticks and the 2Fo-Fc map was contoured at 1.0 σ level. (TIF) [file ppat.1008394.s007.tif]

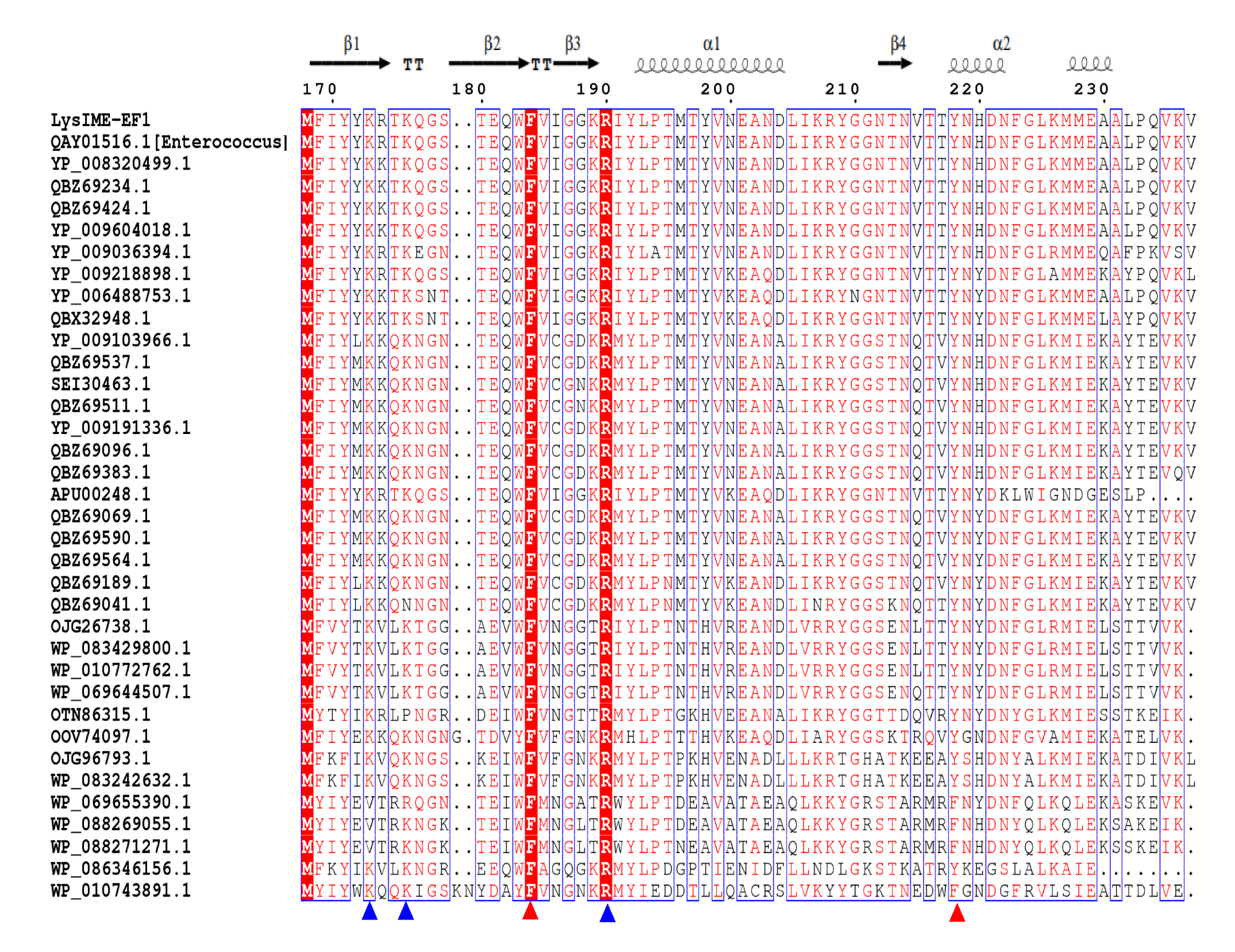

Supplement: S8 Fig — Multiple sequence alignment of LysIME-EF1 CBD and CBD of other endolysins of E. faecalis phages produced by ClusterW (https://www.genome.jp/tools-bin/clustalw) and ESpript 3 (http://espript.ibcp.fr/ESPript/ESPript/). Every 10 residues are indicated with a dot (.) shown above the sequences. Strictly conserved residues are indicated in white on a red background. The blue triangles indicate the positive residues involved in putative cell-wall binding and the red triangles indicate the residues responsible for the oligomerization of the CBD. (TIF) [file ppat.1008394.s008.tif]

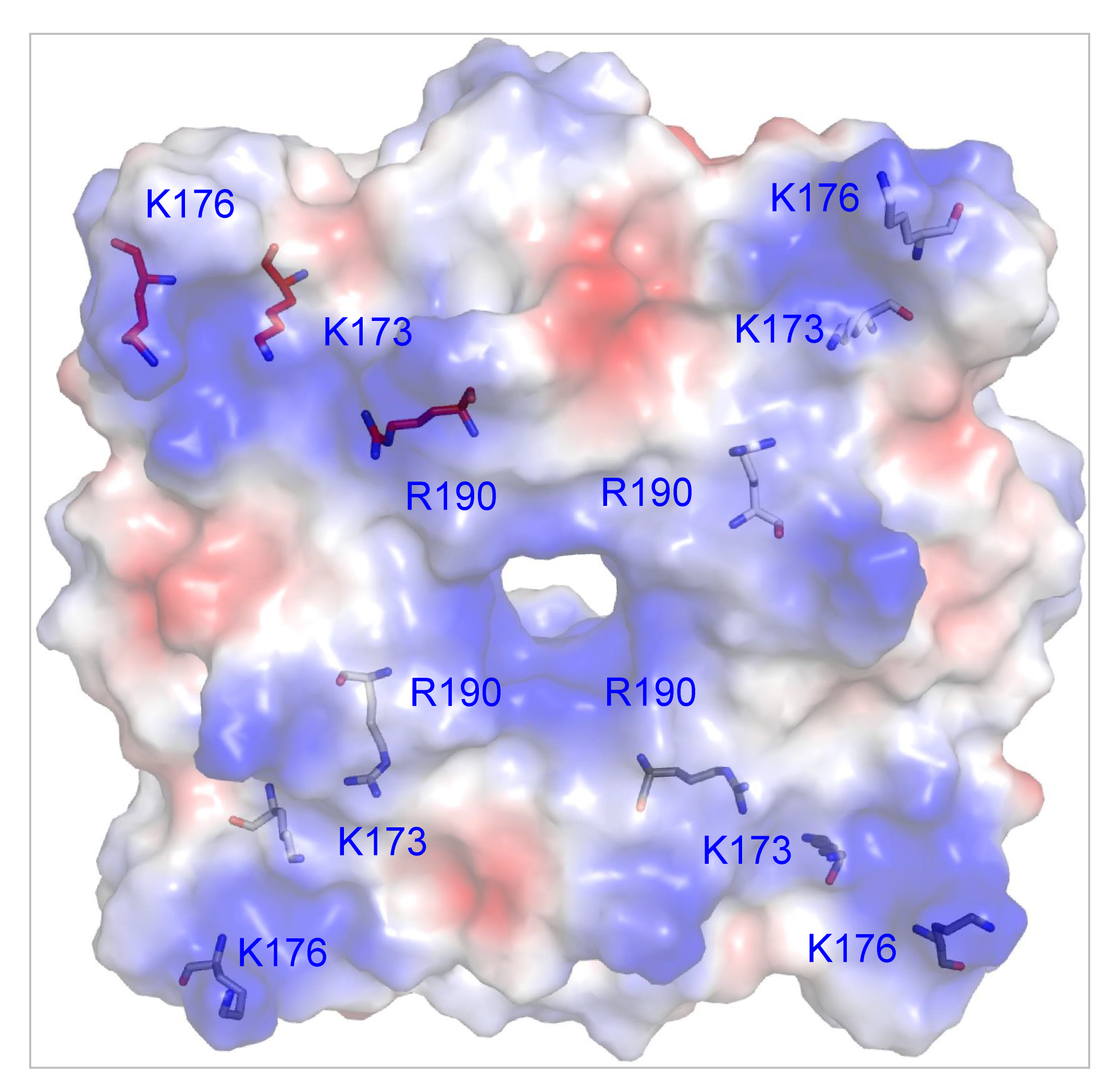

Supplement: S9 Fig — The positively charged patch is located on the planar surface of the LysIME-EF1 CBD tetramer. The putative cell binding residues K173, K1746 and R176 are shown in sticks. Positively and negatively charged regions are colored in blue and red, respectively. (TIF) [file ppat.1008394.s009.tif]

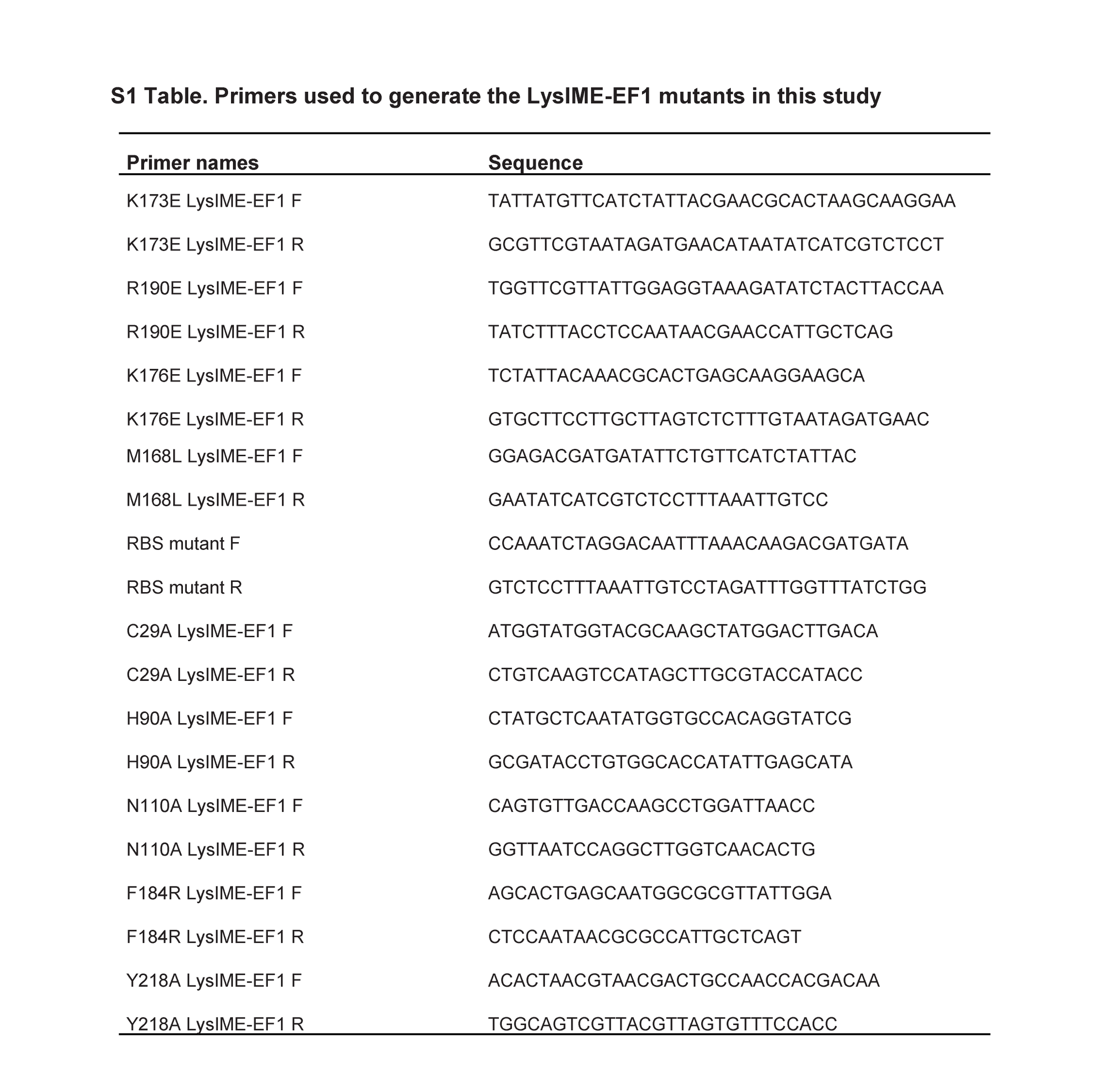

Supplement: S1 Table — (TIF) [file ppat.1008394.s010.tif]
