# Supplementary material for: Structural and functional insights into a novel two-component endolysin encoded by a single gene in Enterococcus faecalis phage
Source: PLoS Pathog. 2020 Mar 16;16(3):e1008394. doi: 10.1371/journal.ppat.1008394 (PMC7098653; doi:10.1371/journal.ppat.1008394)
Supplement: Attachment — Submitted filename: This is an interesting manuscript about a multimeric bacteriophage endolysin.docx [file ppat.1008394.s011.docx]

This is an interesting manuscript about a multimeric bacteriophage endolysin. Phage endolysins are peptidoglycan hydrolases that rapidly destroy the bacterial cell wall. They therefore represent an alternative to conventional antibiotics. The vast majority of endolysins are single proteins/single gene products. They have a catalytic domain (CD) responsible for hydrolytic cleavage of a susceptible peptidoglycan bond and a cell wall binding domain (CBD) responsible for binding to the bacterial surface in a species-specific manner due to unique interactions with wall teichoic acids. Multimeric endolysins, one of which is described in this manuscript, are not as common, but there are a few examples described in the literature.

Some background on multimeric endolysins relevant to this manuscript: PlyC was the first described multimeric endolysin. It is specific to streptococci and it contains two genes/gene products that assemble into an 1:8 ratio yielding 9 subunits in the holoenzyme. Its multimeric nature was first described in 2006 (Nelson et al 2006, PNAS) and its crystal structure was described in 2012 (McGowan et al 2012, PNAS). In a 2014 Plos Pathogens manuscript, Dunne et al. described the second example of a multimeric endolysin. In this manuscript, the authors studied two clostridial endolysins, CTP1L and CD27L, providing crystal structures of a heterotetramer, i.e., a full-length enzyme containing both a CD and a CBD and three copies of the CBD only. The authors contended that the heterotetramer resulted from autoproteolytic degradation of the full-length enzyme to generate the extra CBDs observed in the holoenzyme, although they never provided any experimental evidence to support this contention. A few months later, Proenca et al. in a 2015 Mol Micro paper described an enterococcal endolysin, Lys170, that was likewise a heterotetramer (i.e. composed of a full-length enzyme with CD and CBD and three additional CBD only subunits). However, this group showed through elegant crosslinking and point mutagenesis studies that the heterotetramer was derived from an internal ribosome binding site and an alternate start site at the beginning of the CBD. Furthermore, they analyzed the clostridial endolysins from the 2014 Plos Pathogens paper and identified a similar mechanism missed by those authors. Lastly, a bioinformatics search revealed that dozens, if not hundreds, of additional clostridial and enterococcal endolysins likely possess this unique heterotetramer structure due to the common mechanism of an internal ribosome binding site and an alternate start codon at the beginning of the CBD. In a 2016 JBC manuscript, Dunne et al. confirmed that their multimeric clostridial endolysins are indeed generated by an alternate start site as hypothesized by Proenca et al. rather than through autoproteolytic degradation as they previously suggested. Finally, in 2019, Escobedo et al. reported in Viruses a similar multimeric endolysin, LysTP712, utilizing an alternate start site at the beginning of the CBD for a lactococcal phage endolysin. Thus, multimeric endolysins are much more wide-spread than previously thought.

In the current manuscript, the authors crystallize an enterococcal endolysin, LysIME-EF1. They previously cloned, expressed, and characterized this enzyme (Zhang et al., 2013 Plos One). However, in 2013, they thought this enzyme was a typical monomeric endolysin with an N-terminal CD and a C-terminal CBD with a total mass of ~30 kDa. Had they not cropped their SDS-PAGE gel at ~25 kDa in Figure 3B of the 2013 manuscript, they likely would have seen evidence of additional 8 kDa CBDs that would have revealed the true multimeric nature of this enzyme. Nonetheless, in the present submission, they do not crop their SDS-PAGE gels (Fig. 1B) and do observe these extra CBDs, which are also obviously present in the crystal structure. The crystal structure reveals a heterotetramer similar to those discussed above (i.e. a full length enzyme with three additional CBDs). The structural characteristics are very similar to other multimeric endolysins and/or known individual endolysin domains. Characterization of the heterotetramer by analytical ultracentrifugation and gel filtration as well as site-directed mutagenesis studies of the internal ribosome binding site, the alternate start site, and the active site residues follows point by point from the methods used in the aforementioned manuscripts to characterize heterotetramer endolysins. Thus, while the overall amount of work performed is impressive and these results contribute to an aggregate sum of knowledge for both endolysins, and specifically multimeric endolysins, most of the characterization is perfunctory and there is nothing in these results that is noteworthy or provides new mechanistic insights into this class of enzyme.

The above being said, the authors did some additional studies in this manuscript above and beyond the work done in the previous manuscripts investigating multimeric endolysins. They generated mutants that disrupted the subunit interactions between the CBD tetramer, which had never been attempted before. Additionally, they characterized residues within the CBD important for binding the bacterial surface. Lastly, the authors benchmarked all mutants against the wild type enzyme in an animal model of enterococcal infection. Whether these additional experiments raise the overall profile of the presented work to a higher level of novelty or not is subjective to each reviewer/editor. Regardless, there are numerous errors, misstatements, and incomplete analysis that would first need to be addressed. These issues are presented below as they appear in the manuscript:

Specific comments:

- In general, the manuscript is reasonably well written, but it should be further proof-read by a native English speaker. Many singular/plural errors, subject/verb agreements errors, etc.
- Lines 40-42. It is incorrect to say this is the first structural and biochemical characterization of a multimeric endolysin from an E. faecalis phage. Lys170 was characterized in detail in 2015.
- Lines 70-72. Not all endolysins contain a CD and CBD as indicated. Some only contain a CD. Some contain multiple CDs and/or CBDs, etc.
- Lines 95-96. It is stated that reference 32 proved a truncated CBD was vital to regulate LysIME-EF1 activity. This is very inaccurate. Reference 32 missed the entire point that independent CBDs were even produced by LysIME-EF1.
- Line 110-112. The 8 kDa band was not observed in the previous work because the authors chose to crop the gel in such a manner that an 8 kDa band could not have been observed.
- Lytic activity. All “activity” measurements were based on a turbidity reduction assay in a spectrophotometer. While this type of assay is commonly used in the endolysin field, it is recognized for its limitations….at best, it is an indirect assay of cell lysis based on a drop in optical density due to light scattering. It is more qualitative than quantitative as no direct bactericidal activity is actually measured and light scattering can be severely affected by cell concentration, thickness of the cell wall, buffer components, etc. Additionally, as cells begin to lyse, the buffer become more osmoprotective further skewing the rate of additional lysis. Therefore, most endolysin manuscripts also contain some sort of quantitative assay for bactericidal/antimicrobial activity such as determining log-fold killing or determining the minimal inhibitory concentration. The fact that the authors of the current manuscript did not do anything other than a turbidity reduction assay measured every 20 minutes to compare constructs/mutants is a severe limitation of the current manuscript.
- Lytic assays, part 2. The error bars on the turbidity reduction assays are exceedingly close together. How were statistics performed? It isn’t stated many of these experiments. Were replicates used? If so, were these technical replicates (which seems to be the case given the small deviations in error) or truly independent experimental replicates. It is quite unsettling that the progress curves for the wild-type enzyme in 1C, 1D, 3E, 4D, 5C, and 6C all look extremely similar. In fact, 1C and 3E appear superimposable down to the error bars as do the wild-type curves for 5C and 6C. Better explanation of the experiments, independence, and associated statistics are needed in the methods and the figure legends for all of these. This comment can actually be extended to any figure where statistical data is presented.
- Fig. S2. Were standards run to calibrate the gel filtration? If so, please explain. Zero methods are given anywhere in the manuscript for this work. A figure of a single peak provides very little information without this context. Also, molecular mass is determined, not molecular weight (lines 119, 120, and other places).
- Line 130 and Fig. 1A. The “CHAP” domain is never defined as an acronym or is it explained that the CHAP domain is the catalytic domain (CD) of LysIME-EF1. It is never defined in the introduction, the results, the methods, or the figure legend for 1A. This should be addressed.
- Line 130-131. Since the authors are already making extensive use of supplemental material, they should provide a purification gel for the CHAP and CBD domains….actually for all mutants…see below.
- Line 132 and throughout the manuscript: The authors use an indirect light scattering assay to measure turbidity reduction of a bacterial culture. While it indirectly may relate to a bacteriolytic event, it in no way is a bactericidal assay, which has a specific definition related to a decrease in colony forming units. Please to do use the term bactericidal (used 14 times in the current manuscript), when no bactericidal assays were performed.
- All mutants. Numerous mutants and domain constructs are made throughout the manuscript….active site mutants, start codon mutants, binding site mutants/chimeras, subunit interactions mutants, etc. However, the authors do not present a single gel to show that any of these are soluble, properly folded, or pure. In lieu of a purification gel, did the authors do any biophysical experiments to demonstrate proper folding of the mutants (i.e. gel filtration, circular dichroism, etc.)? When a given mutant has a dramatic phenotype, it is important to demonstrate that the observed phenotype is the results of the mutant and not an artefact of misfolding, etc. None of these types of experiments were done in this manuscript.
- Figure 1C. Many issues. First, it is stated that 1 uM of the wild-type enzyme was used, but there is no indication anywhere (results, methods, figure legends) about how much CHAP or CBD was used. Were these equimolar amounts? If not, how much was used? Second, Line 134 indicates that there was no activity even at 100 ug/ml. This suggests different amounts of CBD and/or CHAP were used in 1C, but this is not the case, so something is very wrong with this sentence. Also, reporting of concentration should be consistant…i.e. how does 100 ug/ml relate to 1 uM? Third, the authors state that the CHAP, CBD, and CHAP/CBD did not have activity, but there is clearly activity greater than PBS control in the graph, so this statement is incorrect. Did the authors do any statistics to compare the levels of “activity”? Third, how do the authors explain that CBD appears to have greater “activity” than the PBS control when the CBD alone possesses no catalytic domain or active site residues? What mechanism is proposed for this observation? Could the “activity” be an artifact of cells clumping together due to the binding domain? If so, actual bactericidal assays should have been performed. What mechanism is proposed for the enhancement of activity of CHAP + CBD?
- Line 137-138. The authors contend that the CBD regulates the lytic activity of LysIME-EF1. How so? They have shown that addition of an excess amount of pre-formed CBDs has an inhibitory effect, presumably by competing for binding epitopes, but that is not the same thing as having the CBD regulate its catalytic domain.
- Crystallography. It appears as if the authors had two different diffracting crystals. One (space group P12_1_1) contained the heterodimer and one (space group I422) contained just a tetramer of the CBD, even though both crystals were produced from wild-type enzyme. Is this the case, because it is not clear in the written methods? Did the authors ever crystallize from CBD-only constructs?
- Line 158. Why do the authors say extra CBDs were “unexpectedly” built into the final model? They knew this was a multimeric enzyme based on all the evidence in the literature, published structures, and their own data. This should not have been unexpected.
- Line 160-161. Why do the authors regard one particular side to be responsible for recognition of the peptidoglycan? Why not the other side? Do they have experimental evidence or other rationale for making this statement or is it just an assumption?
- Database entries. PDB and/or GenBank accession numbers should be provided for all sequences. For example, nothing is stated for LysK.
- Line 171. In Figure 3B, which residues constitute the catalytic triad and which form the calcium binding site? Details are continually lacking in the results text and never present in the figure legends. As a general rule, all figure legends, and in particular the supplementary figure legends, do not contain enough information to adequately explain the displayed figure.
- Line 187-188. Is this data not shown?
- Figure 4B. The color coding of the residue numbers and the side chains seems to be off as this figure is very confusing in context of the results presented in lines 192-195. Also, A23 should be A231 in line 194.
- Line 203. Does 18.7 ml correspond to a monomer? Again, where standards used? No methods.
- Line 214. Post-incubation is the correct term, not post-infection.
- Figure 5C. Again, what were the concentrations of the proteins used for these experiments? Nothing stated in results, figure legends, etc.
- Figure 7. The 190E mutant is not visualized well on the graph. Also, it is stated that the authors did in vivo experiments with K173 and K176. Why were these results not presented?
- Did the authors attempt to do any statistical analysis for the in vivo data?
- Line 278. It is stated that the data is not shown for infected mice not administered LysIME-EF1 in Figure 7. However, a control is shown in the figure. What is the control if it is not infected mice not administered LysIME-EF1?
- Line 295. “The structure of native LysIME-EF1, which is distinct among endolysins”…actually, it is not very distinct. The authors themselves state the high similarity to known endolysins via Dali searches and RMS calculations. Additionally, the heterotetramer quaternary structure is well described in the literature.
- Line 299-304. “…suggesting that the integrity of the structure can be maintained only in the presence of the three additional truncated CBDs.” Actually, there is no data that would support comments about structural integrity one way or another. The authors only show that the enzyme requires a multimeric assembly for maximal activity…this has nothing to do with structural integrity or stability.
- Line 304-306. “We can further argue that, without the extra CBD fragments, the CBD of the full-length LysIME-EF1 would not be anchored in an optimal orientation to bind to the cell wall and carry out its function.” No, actually, you can’t say that. The authors have provided no evidence of enzyme kinetics, no evidence of binding constants/affinities between wild type and mutants, no co-crystals of LysIME-EF1 and cell wall epitopes/substrates to justify any such statements about anchoring to the bacterial surface or optimal orientation, even in a speculative discussion.
- Line 311-314. The authors forgot about the work on PlyC….a multimeric endolysin not mentioned.
- In general, the methods section is very brief and not adequate. Some sections are entirely missing (i.e. analytical gel filtration, etc.)
