# Supplementary material for: Structural and functional insights into a novel two-component endolysin encoded by a single gene in Enterococcus faecalis phage
Source: PLoS Pathog. 2020 Mar 16;16(3):e1008394. doi: 10.1371/journal.ppat.1008394 (PMC7098653; doi:10.1371/journal.ppat.1008394)
Supplement: Attachment — Submitted filename: Responses to PPATHOGENS-D-19-01755.docx [file ppat.1008394.s012.docx]

**Point-by-point responses to Reviewer’s comments:**

**Reviewer #1**

Zhou and coworkers report the first structure of a known E. faecalis-targeted endolysin, LysIME-EF1 and find it posing multimeric composition, i.e., a full-length endolysin with three extra CBDs. A lysin (Lys170, the endolysin from enterococcal phage F170/08) with similar composition has been reported by reference 28 (Proenca D, Velours C, Leandro C, Garcia M, Pimentel M, et al. A two-component, multimeric endolysin encoded by a single gene. Mol Microbiol 2015，95: 739-753). The difference is that the current finding is based on the crystallographic study of LysIME-EF1, which is important for further understanding the action model of similar lysins against E. faecalis.

**Major Issues:**

1. Fig 1B and Fig 5B. The images show that the band intensity of the CBD (8 kD) is much lighter compared with that of the full length 30 kD LysIME-EF1. How to explain this result if it is correct that the purified LysIME-EF1 consists of a full-length of 30 kD LysIME-EF1 and three extra 8 kD of CBDs? In theory, In the concentration of the CBD should be 3 times of that of the full length 30 kD LysIME-EF1 during SDS-PAGE electrophoresis. For instance, both bands of Lys170 in the SDS-PAGE showed similar density in the previous report (Ref. 20).

Authors response: To explain this result, we performed the experiment again, and the figures are now much improved and obvious (Please see the figures below, left panel). The band intensity of LysIME-EF1 CBD is a little lighter, and the reason may be the following:

Firstly, the brightness of the 30 kDa band and the 8 kDa is related to their mass values, which depends on the number of amino acids in the protein. In our structure, there is one molecule with full-length LysIME-EF1 and three CBD molecules of LysIME-EF1, and the mass ratio is 1:0.88.

Secondly, a phenomenon called smearing which is a usual case concerning the quality of gel and samples, and when the proteins with small molecular mass, such as Ubiquitin, are run during SDS-PAGE. Of course, if we look carefully at the band, the shadow of LysIME-EF1 CBD is distinctively visible, and a similar result can be observed in Lys170, which is showed as below (Please see the figures below, right panel).

10

15

25

35

45

55

75

100

LysIME-EF1

Maker

k Da

(Exacted from Proenca D, Velours C, Leandro C, Garcia M, Pimentel M, et al. (2015) A two-component, multimeric endolysin encoded by a single gene. Mol Microbiol 95: 739-753)

(Exacted from our experimental data)

2. Fig 5C: Additional experiments should be done by mixing M168L/RBS LysIME-EF1 mutant with different ratio of the purified CBD to see if the lytic activity could be restored in order to confirm the multimeric composition.

Authors response: We conducted the experiment as suggested. However, we found that by mixing M168L/RBS mutant at a different ratio of purified LysIME1 CBD (res168-237), very low lytic activity was observed (Please see the figures below, left panel). The data have been shown in Fig. 5D in our manuscript.

In addition, we noticed that LysIEM-EF1 M168L/RBS mut exists as a stable homo-tetramer (Please see the figures below, right panel). The structure determined in this study showed that the interaction among the CBDs is quite strong. Besides, another study published recently possesses a similar result with us (Escobedo S, Campelo AB, Wegmann U, Garcia P, Rodriguez A, et al. (2019) Insight into the Lytic Functions of the Lactococcal Prophage TP712. Viruses 11. **Fig. S2A and S2B**).

**Control**

**WT**

**M168L&RBS mut ：CBD 1:1**

**M168L&RBS mut ：CBD 1:3**

**M168L&RBS mut ：CBD 1:9**

(Exacted from our experimental data)

(Exacted from our experimental data Fig 5D)

3. Fig 1C, 3E and 6C: From the lysis curves of the mutants, it is hard to judge the activity difference between them. It is suggested to use the more sensitive culture methods, such as plate counting, to confirm the activity of different mutants, especially K173E, K176E, C29A, H90A, N110A, CHAP, CBD, and CHAP+CBD.

Authors response: In the current revised manuscript, we used the method of the plate counting that suggested by the referee to compare the lytic activity among wild type LysIME-EF1 and other mutants. Now, the results clearly show distinct difference of their lytic activity in the current version of the manuscript. Please See Fig. 1C, 1D, 3E, 4D, 5C and 5D.

4. Fig 7 and all related text: The in-vivo infection model might just prove that the protection rates are related to the activity of the lysin used, which did not add more information to the structure of LysIME-EF1 and could be deleted.

Authors response: The in-vivo infection model was used to demonstrate that the key residues such as the amino acids involved in *E. faecalis* binding are also important in vivo. Although the bactericidal activity assays were performed in vitro, in order to make the research more integrity, the section was not deleted. However, the figure was modified to make it clearer. Please see Fig. 7 in the new manuscript.

**Minor Issues:**

1. Page 2, lines 27-2: Most natural endolysins has limited host range, but there are several lysins with extended killing spectra, for instance, chimeric lysin ClyR, natural lysin PlySs2 and PlyV12. What does low persistence mean? Please rephase this sentence.

Authors response: We want to show that the limitation of endolysin application is due to its narrow spectrum of activity. The “low persistence” may not be a good expression. In the new manuscript, we edited the manuscript as “However, their application is still hindered by their narrow spectra of activity” (please see line 27-28). We also find that the engineered chimeric endolysins are good examples to extend their lytic activity. The related sentence has been rephrased, and we have cited the related literature in the new version of the manuscript (please see the references 23 and 24).

2. Page 4, lines 59: Enterococcus faecalis should be written as E. faecalis after appearing the first time. Correct this throughout the text.

Authors response: We have corrected it in the new version of the manuscript.

1. Line 10, page 187, Supporting figure is missing.

Authors response: As the referee suggested, now the data is added as Fig. S6A.

2. Page 12, line 214：Interaction of lysin with bacterial cells is not infection. Post-infection could be changed to post-addition. The same mistake also goes to page 13, line 239.

Authors response: This is an expression error. In the new manuscript, we have deleted it (please see line 206-208). Thank you again.

5. Page 12, line 216: What does “ability” mean?

Authors response: We are sorry for the poor preparation. It was corrected as “activity” in the new version of the manuscript now (please see line 206).

6. Page 15, line 277: 10 hours post infection? In the Figure 7 legend and the material method, it is mentioned that lysins were administrated 1 h post infection.

Authors response: We are sorry for the writing error. In the new manuscript, we have corrected it to “1 hour after post-infection” (please see line 274).

**Reviewer #2:**

Lysins are hydrolytic enzymes produced by bacteriophages in order to cleave the host’s cell wall and are considered as a potential strategy for treatment of bacterial infections caused by multidrug-resistant pathogenic microorganisms. This study focuses on LysIME-EF1, an endolysin that specifically lyses Enterococcus faecalis, a major pathogen with high levels of antibiotic resistance.

In this study, the authors observed that expression of LysIME-EF1 results in two polypeptides, a full length product and a C-terminal CBD domain. Both polypeptides are necessary for the assemly of the functional lysin. They further determined the crystal structure of LysIME-EF1 at a high resolution. The structure revealed an asymmetric assembly containing one full length LysIME-EF1 and three CBD domains. Based on the structure, the authors identified potential key residues, designed mutants and assessed their cell binding capability. Overall, the study constitutes a novel insight into this therapeutically important enzyme and open the possibilities for the future structure-based design of lysins with higher activity and broader antimicrobial spectrum.

**Minor Issues:**

1. **The author reported that one full length LysIME-EF1 and three CBD domains co-assemble to a functional lysin. Is this the only possible** **assembly? Does the CHAP domain block the additional CHAP domain to assemble on a CBD tetramer?**

Authors response: We thank the referee for pointing this out. In this study, we identified a multimeric endolysin and determined the crystal structure of the holoenzyme and the homotetrameric CBD, respectively. The structure enabled us to see that the holoenzyme is composed of full-length CBD (Red part in Fig. 2A) and three truncated CBDs (Gray part in Fig. 2A). Also, we proved that the three truncated CBDs were produced through alternative translation initiation and the unique structure of the holoenzyme LysIME-EF1 is related to its highly efficient lytic activity. Based on the structure present and our biochemical study, only LysIME-EF1 assembles to functional unit with full-length LysIEM-EF1 and three CBDs, can it be activated.

From the structure presented here, it is clear that functional unit of LysIME-EF1 contains three truncated CBDs and a full-length LysIME-EF1, there is only one CHAP domain and it does not interact with any CBD of LysIME-EF1, so the CHAP domain is believed not to be involved in blocking the additional CHAP domain to assemble the CBD tetramer.

2. **The authors reported that the CHAP alone, CBD alone or a mixture of the two constructs, exhibited no bactericidal activity. This indicates that 1) C****HAP domain and CBD tetramer can not co-assemble in vitro. 2) assembly with the CBD tetramer likely has an allosteric effect on the CHAP domain. The structural comparison of CHAP domain alone and that in complex with CBD will be helpful.**

Authors response: In our study, we investigated the bactericidal activity of the CHAP domain and the CBD domain, because some endolysins are only composed of catalytic domains. In our study, and the mixture of the two domains exhibited no bactericidal activity, we believe this may because that the two domains have no interaction, which is obvious from the structure that clearly shows no interaction.

1) Agreed, the CHAP domain and CBD tetramer can’t co-assemble in vitro because there is no interaction between the CHAP domain and CBD tetramer.
2) From the structure of the holoenzyme, there is no allosteric effect between the CBD tetramer and the CHAP domain.

**3. The presence of secondary** **translation products has been observed previously in other proteins, such as MthK (Jiang et al, Nature, 2002) and Tvok (Parfenova et al, J Biol Chem, 2007).**

Authors response: We thank the referee for the appreciation for our paper. Agreed, the presence of secondary translation has widely existed. However, there are few reports on endolysins except the examples we cited. Importantly, the structures are rarely because the fact that it is extremely difficult in getting the crystals due to the flexibility results from the lack of the interaction between the two domains.

**Reviewer #3**

Some background on multimeric endolysins relevant to this manuscript: PlyC was the first described multimeric endolysin. It is specific to streptococci and it contains two genes/gene products that assemble into an 1:8 ratio yielding 9 subunits in the holoenzyme. Its multimeric nature was first described in 2006 (Nelson et al 2006, PNAS) and its crystal structure was described in 2012 (McGowan et al 2012, PNAS). In a 2014 Plos Pathogens manuscript, Dunne et al. described the second example of a multimeric endolysin. In this manuscript, the authors studied two clostridial endolysins, CTP1L and CD27L, providing crystal structures of a heterotetramer, i.e., a full-length enzyme containing both a CD and a CBD and three copies of the CBD only. The authors contended that the heterotetramer resulted from autoproteolytic degradation of the full-length enzyme to generate the extra CBDs observed in the holoenzyme, although they never provided any experimental evidence to support this contention. A few months later, Proenca et al. in a 2015 Mol Micro paper described an enterococcal endolysin, Lys170, that was likewise a heterotetramer (i.e. composed of a full-length enzyme with CD and CBD and three additional CBD only subunits). However, this group showed through elegant crosslinking and point mutagenesis studies that the heterotetramer was derived from an internal ribosome binding site and an alternate start site at the beginning of the CBD. Furthermore, they analyzed the clostridial endolysins from the 2014 Plos Pathogens paper and identified a similar mechanism missed by those authors. Lastly, a bioinformatics search revealed that dozens, if not hundreds, of additional clostridial and enterococcal endolysins likely possess this unique heterotetramer structure due to the common mechanism of an internal ribosome binding site and an alternate start codon at the beginning of the CBD. In a 2016 JBC manuscript, Dunne et al. confirmed that their multimeric clostridial endolysins are indeed generated by an alternate start site as hypothesized by Proenca et al. rather than through autoproteolytic degradation as they previously suggested. Finally, in 2019, Escobedo et al. reported in Viruses a similar multimeric endolysin, LysTP712, utilizing an alternate start site at the beginning of the CBD for a lactococcal phage endolysin. Thus, multimeric endolysins are much more wide-spread than previously thought.

In the current manuscript, the authors crystallize an enterococcal endolysin, LysIME-EF1. They previously cloned, expressed, and characterized this enzyme (Zhang et al., 2013 Plos One). However, in 2013, they thought this enzyme was a typical monomeric endolysin with an N-terminal CD and a C-terminal CBD with a total mass of ~30 kDa. Had they not cropped their SDS-PAGE gel at ~25 kDa in Figure 3B of the 2013 manuscript, they likely would have seen evidence of additional 8 kDa CBDs that would have revealed the true multimeric nature of this enzyme. Nonetheless, in the present submission, they do not crop their SDS-PAGE gels (Fig. 1B) and do observe these extra CBDs, which are also obviously present in the crystal structure. The crystal structure reveals a heterotetramer similar to those discussed above (i.e. a full length enzyme with three additional CBDs). The structural characteristics are very similar to other multimeric endolysins and/or known individual endolysin domains. Characterization of the heterotetramer by analytical ultracentrifugation and gel filtration as well as site-directed mutagenesis studies of the internal ribosome binding site, the alternate start site, and the active site residues follows point by point from the methods used in the aforementioned manuscripts to characterize heterotetramer endolysins. Thus, while the overall amount of work performed is impressive and these results contribute to an aggregate sum of knowledge for both endolysins, and specifically multimeric endolysins, most of the characterization is perfunctory and there is nothing in these results that is noteworthy or provides new mechanistic insights into this class of enzyme.

The above being said, the authors did some additional studies in this manuscript above and beyond the work done in the previous manuscripts investigating multimeric endolysins. They generated mutants that disrupted the subunit interactions between the CBD tetramer, which had never been attempted before. Additionally, they characterized residues within the CBD important for binding the bacterial surface. Lastly, the authors benchmarked all mutants against the wild type enzyme in an animal model of enterococcal infection. Whether these additional experiments raise the overall profile of the presented work to a higher level of novelty or not is subjective to each reviewer/editor. Regardless, there are numerous errors, misstatements, and incomplete analysis that would first need to be addressed. These issues are presented below as they appear in the manuscript:

**Specific comments:**

- In general, the manuscript is reasonably well written, but it should be further proof-read by a native English speaker. Many singular/plural errors, subject/verb agreements errors, etc.

Authors response: Agreed. The manuscript has been edited for clarity and readability. Particular attention has been paid to the grammar as well as the use of words and phrases while revising the current version of the manuscript.

- Lines 40-42. It is incorrect to say this is the first structural and biochemical characterization of a multimeric endolysin from an E. faecalis phage. Lys170 was characterized in detail in 2015.

Authors response: We edited the manuscript as “In this study, a multimeric endolysin was biochemically characterized and their structure was determined. To our knowledge, it is the first determined structure of multimeric endolysin encoded by a single gene in *E. faecalis* phages” (please see line 39-42). As such, it may provide valuable insights into designing potent endolysins against the opportunistic pathogen *E. faecalis*. (Dunne M, Leicht S, Krichel B, Mertens HD, Thompson A, et al. (2016) Crystal Structure of the CTP1L Endolysin Reveals How Its Activity Is Regulated by a Secondary Translation Product. J Biol Chem 291: 4882-4893.). However, LysIME-EF1 displays significant difference in its structure with CTP1L.

- Lines 70-72. Not all endolysins contain a CD and CBD as indicated. Some only contain a CD. Some contain multiple CDs and/or CBDs, etc.

Authors response: In the current version of the manuscript we stated as “Generally, most endolysins are composed of a single polypeptide chain divided into an N-terminal catalytic domain (CD), a C-terminal cell wall binding domain (CBD), which are interconnected by a short flexible linker. Despite some endolysins contains only a CD and some endolysins are composed of multiple CDs linked to CBD” (please see line 64-68).

- Lines 95-96. It is stated that reference 32 proved a truncated CBD was vital to regulate LysIME-EF1 activity. This is very inaccurate. Reference 32 missed the entire point that independent CBDs were even produced by LysIME-EF1.

Authors response: We are sorry for the mistake. A corrected version is now in the revised manuscript.

- Line 110-112. The 8 kDa band was not observed in the previous work because the authors chose to crop the gel in such a manner that an 8 kDa band could not have been observed.

Authors response: Yes, in the beginning, the 8 kDa band was ignored in our previous work because of relatively long running time for SDS-PAGE.

- Lytic activity. All “activity” measurements were based on a turbidity reduction assay in a spectrophotometer. While this type of assay is commonly used in the endolysin field, it is recognized for its limitations….at best, it is an indirect assay of cell lysis based on a drop in optical density due to light scattering. It is more qualitative than quantitative as no direct bactericidal activity is actually measured and light scattering can be severely affected by cell concentration, thickness of the cell wall, buffer components, etc. Additionally, as cells begin to lyse, the buffer become more osmoprotective further skewing the rate of additional lysis. Therefore, most endolysin manuscripts also contain some sort of quantitative assay for bactericidal/antimicrobial activity such as determining log-fold killing or determining the minimal inhibitory concentration. The fact that the authors of the current manuscript did not do anything other than a turbidity reduction assay measured every 20 minutes to compare constructs/mutants is a severe limitation of the current manuscript.

Authors response: In the current manuscript, we compared the bactericidal activity of the constructs/mutants using plate counting. Now the results are much clearer than the previous version of the manuscript.

Lytic assays, part 2. The error bars on the turbidity reduction assays are exceedingly close together. How were statistics performed? It isn’t stated many of these experiments. Were replicates used? If so, were these technical replicates (which seems to be the case given the small deviations in error) or truly independent experimental replicates. It is quite unsettling that the progress curves for the wild-type enzyme in 1C, 1D, 3E, 4D, 5C, and 6C all look extremely similar. In fact, 1C and 3E appear superimposable down to the error bars as do the wild-type curves for 5C and 6C. Better explanation of the experiments, independence, and associated statistics are needed in the methods and the figure legends for all of these. This comment can actually be extended to any figure where statistical data is presented.

Authors response: Indeed, in our previous manuscript, we compared the lytic activity by performing turbidity reduction assays. As described in the paper, the lytic activity is quite high, and it is hard to measure the OD_600_ accurately. Even sometimes, when the OD_600_ is measured, the values are quite similar leading to the error bars which are close together. Now in the current version of the manuscript, we use a more direct and a clear method of plate counting, and now the data is reasonable.

- Fig. S2. Were standards run to calibrate the gel filtration? If so, please explain. Zero methods are given anywhere in the manuscript for this work. A figure of a single peak provides very little information without this context. Also, molecular mass is determined, not molecular weight (lines 119, 120, and other places).

Authors response: Standard proteins were run in the current manuscript, and the method section was also added (please see line 397-405). The data were shown in Fig. S2A, S3A and S6A. We used the word “molecular weight” by mistake, now it is corrected in the current version of the manuscript (please see line 115 and 118).

- Line 130 and Fig. 1A. The “CHAP” domain is never defined as an acronym or is it explained that the CHAP domain is the catalytic domain (CD) of LysIME-EF1. It is never defined in the introduction, the results, the methods, or the figure legend for 1A. This should be addressed.

Authors response: A corrected version is now in the revised manuscript. Now, we have addressed this correction in the manuscript as “In our previous work, we isolated an endolysin derived from *E. faecalis* bacteriophage. PSI-BLAST search predicted that LysIME-EF1 consists of an N-terminal catalytic CHAP (cysteine, histidine-dependent amidohydrolases/peptidases) domain and a CBD (C-terminal cell wall binding domain)” (please see line 99-102).

- Line 130-131. Since the authors are already making extensive use of supplemental material, they should provide a purification gel for the CHAP and CBD domains….actually for all mutants…see below.

Authors response: The method and purification gel to purify the CHAP and the CBD are added in the current version of the manuscript as Fig. S3A, S3B and S6A.

- Line 132 and throughout the manuscript: The authors use an indirect light scattering assay to measure turbidity reduction of a bacterial culture. While it indirectly may relate to a bacteriolytic event, it in no way is a bactericidal assay, which has a specific definition related to a decrease in colony forming units. Please to do use the term bactericidal (used 14 times in the current manuscript), when no bactericidal assays were performed.

Authors response: In the current version of the manuscript, we used plate counting method to compare the bactericidal activity of wild type LysIME-EF1 and constructs/mutants. Now, It’s more sensitive than the previous method. Thank you again.

- All mutants. Numerous mutants and domain constructs are made throughout the manuscript….active site mutants, start codon mutants, binding site mutants/chimeras, subunit interactions mutants, etc. However, the authors do not present a single gel to show that any of these are soluble, properly folded, or pure. In lieu of a purification gel, did the authors do any biophysical experiments to demonstrate proper folding of the mutants (i.e. gel filtration, circular dichroism, etc.)? When a given mutant has a dramatic phenotype, it is important to demonstrate that the observed phenotype is the results of the mutant and not an artefact of misfolding, etc. None of these types of experiments were done in this manuscript.

Authors response: In the current version of the manuscript, the gel filtration files, as well as the SDS-PAGE, are added as Fig. S2A, S3A and 3B, Fig. S6B.

- Figure 1C. Many issues. First, it is stated that 1 uM of the wild-type enzyme was used, but there is no indication anywhere (results, methods, figure legends) about how much CHAP or CBD was used. Were these equimolar amounts? If not, how much was used?

Authors response: We are sorry for the poor presentation. In the experiment, all the proteins are used the equimolar concentrations (0.1 μM) and we also checked throughout the current version of the manuscript and clearly addressed it.

- Second, Line 134 indicates that there was no activity even at 100 ug/ml. This suggests different amounts of CBD and/or CHAP were used in 1C, but this is not the case, so something is very wrong with this sentence. Also, reporting of concentration should be consistant…i.e. how does 100 ug/ml relate to 1 uM?

Authors response: We intended to address that only the wild type LysIME-EF1 possess the lytic activity and that the CBD alone or the CHAP alone can’t lyse *E. faecalis.* In the current version of the manuscript, we used μM instead of μg/ml (please see line 128).

- Third, the authors state that the CHAP, CBD, and CHAP/CBD did not have activity, but there is clearly activity greater than PBS control in the graph, so this statement is incorrect. Did the authors do any statistics to compare the levels of “activity”? Third, how do the authors explain that CBD appears to have greater “activity” than the PBS control when the CBD alone possesses no catalytic domain or active site residues? What mechanism is proposed for this observation? Could the “activity” be an artifact of cells clumping together due to the binding domain? If so, actual bactericidal assays should have been performed. What mechanism is proposed for the enhancement of activity of CHAP + CBD?

Authors response: We thank the referee for pointing this out. When we conducted the experiment, we compared the lytic activity between wild type LysIME-EF1 and other mutants. We found that adding the wild type LysIME-EF1 to *E. faecalis,* the cells are lysed quickly, leading to the cells clumping together and this may result in the inaccuracy of the data measured by OD_600_. In the current version of the manuscript, a more precise method (plate counting) was performed and it is now clear that there is little difference among LysIME-EF1 CHAP, LysIME-EF1 CBD and wild type LysIME-EF1+LysIME CBD, respectively. Essentially, we did this to study that whether the CBD is required for its lytic activity or not, and if the CHAP and CBD have interaction or not because some endolysins are only composed of CD. In the end, we discovered that only the wild type LysIME-EF1 could lyse the cells.

- Line 137-138. The authors contend that the CBD regulates the lytic activity of LysIME-EF1. How so? They have shown that addition of an excess amount of pre-formed CBDs has an inhibitory effect, presumably by competing for binding epitopes, but that is not the same thing as having the CBD regulate its catalytic domain.

Authors response: We agree that it is incorrect to state that the CBD regulates the lytic activity. Now, the experiments (Fig. 1C and 1D) are conducted because we want to compare the lytic activity and investigate that if the CBD is the limiting factor for LysIME-EF1. It is a usual experiment in other endolysins such as Lys170 (Proenca D, Velours C, Leandro C, Garcia M, Pimentel M, et al. (2015) A two-component, multimeric endolysin encoded by a single gene. Mol Microbiol 95: 739-753), and CTP1L (Dunne M, Leicht S, Krichel B, Mertens HD, Thompson A, et al. (2016) Crystal Structure of the CTP1L Endolysin Reveals How Its Activity Is Regulated by a Secondary Translation Product. J Biol Chem 291: 4882-4893.6).

- Crystallography. It appears as if the authors had two different diffracting crystals. One (space group P12_1_1) contained the heterodimer and one (space group I422) contained just a tetramer of the CBD, even though both crystals were produced from wild-type enzyme. Is this the case, because it is not clear in the written methods? Did the authors ever crystallize from CBD-only constructs?

Authors response: Correct. The structure of holoenzyme LysIME-EF1 and LysIME-EF1 were determined by different crystals grown in different conditions. We did not crystallize LysIME-EF1 CBD-only constructs. Therefore, we rewrite it again to make it clear in the current version of the manuscript.

- Line 158. Why do the authors say extra CBDs were “unexpectedly” built into the final model? They knew this was a multimeric enzyme based on all the evidence in the literature, published structures, and their own data. This should not have been unexpected.

Authors response: It is an expression error, and we have corrected it as “successfully” in the new version of the manuscript (please see Line 149).

- Line 160-161. Why do the authors regard one particular side to be responsible for recognition of the peptidoglycan? Why not the other side? Do they have experimental evidence or other rationale for making this statement or is it just an assumption?

Authors response: When we analyzed the structure of the holoenzyme, we compared it with PlyC (PDB: 4F88). A similarity between them is that they are composed of a ring-shaped CBD which is responsible for the substrate binding. Besides, the catalytic domain is positioned above the CBDs. It was proved that the side opposite to PlyCA (the bottom of the CBDs) is responsible for cell binding, so we speculated that the planar side of the tetrameric CBDs is responsible for the recognition of the peptidoglycan. Subsequently, it is demonstrated by our experiment that R190E LysIME-EF1 is nearly unable to bind with *E. faecalis* (Fig. 6B).

Database entries. PDB and/or GenBank accession numbers should be provided for all sequences. For example, nothing is stated for LysK.

Authors response: The PDB ID of LysK (PDB: 4CSH) and LysGH15 (PDB: 4olk) are added in the current version of the manuscript (please see line 157-158).

- Line 171. In Figure 3B, which residues constitute the catalytic triad and which form the calcium binding site? Details are continually lacking in the results text and never present in the figure legends. As a general rule, all figure legends, and in particular the supplementary figure legends, do not contain enough information to adequately explain the displayed figure.

Authors response: A corrected version is now in the revised manuscript. They are described as “Analogously to LysGH15, LysIME-EF1 CHAP domain also contains a calcium-binding site and a classical amidohydrolase catalytic triad (C29, H90 and N110) (Fig. 3B). Electron density of the calcium ion is clearly identified in the crystal structure, and the calcium ion is coordinated by the conserved residues D20, D22, W24, G27, D31 and one water molecule (Fig. 3B and 3C)” (please see line 161-166).

- Line 187-188. Is this data not shown?

Authors response: We thank the referee for pointing this out, now the data is added as Fig. S6A.

- Figure 4B. The color coding of the residue numbers and the side chains seems to be off as this figure is very confusing in context of the results presented in lines 192-195. Also, A23 should be A231 in line 194.

Authors response: We are so sorry that the residues numbers are off, as shown in the previous version of the manuscript. Now we change it to a new figure, and A23 is corrected as A231 (please see Fig. 4B and line 187).

- Line 203. Does 18.7 ml correspond to a monomer? Again, where standards used? No methods.

Authors response: In our previous version of the manuscript, the conclusion was made based on the result that the elution profiles of wild type LysIME-EF1 CBD and the mutant are different. In the new version of the manuscript, mixture of standard proteins was run as the referee suggested, which makes it obvious to make the conclusion (please see Fig. S6A).

Line 214. Post-incubation is the correct term, not post-infection.

Authors response: We have edited the manuscript as “The results showed that the antibacterial activity was nearly lost when the interactions between the CBDs were disrupted, suggesting that tetramerization of LysIME-EF1 is necessary for its highly efficient lysis of *E. faecalis* cells” (please see line 206-208).

- Figure 5C. Again, what were the concentrations of the proteins used for these experiments? Nothing stated in results, figure legends, etc.

Authors response: The concentrations of the proteins used for experiments were 0.1 µM. In the new version of the manuscript, we have corrected it as shown in Figure legends.

- Figure 7. The 190E mutant is not visualized well on the graph. Also, it is stated that the authors did in vivo experiments with K173 and K176. Why were these results not presented?

Authors response: In our previous version of the manuscript, the lines were overlapped, making it difficult to distinguish them. Now, we edit the data as a histogram, which is much clearer. K173E and K176E residues are also included in Fig. 7.

- Did the authors attempt to do any statistical analysis for the in vivo data?

Authors response: We repeated each experiment three times, and now the vivo data is shown as Fig. 7

- Line 278. It is stated that the data is not shown for infected mice not administered LysIME-EF1 in Figure 7. However, a control is shown in the figure. What is the control if it is not infected mice not administered LysIME-EF1?

Authors response: We are sorry for the poor presentation, and now it is corrected. As shown in Fig. 7, the 1xPBS buffer is used as a control and the method is showed in line 454.

Line 295. “The structure of native LysIME-EF1, which is distinct among endolysins”…actually, it is not very distinct. The authors themselves state the high similarity to known endolysins via Dali searches and RMS calculations. Additionally, the heterotetramer quaternary structure is well described in the literature.

Authors response: We are sorry for the poor presentation. We mean that the structure of LysIME-EF1 is distinct to the other solved structures of endolysins, and we described it as “The structure of holoenzyme LysIME-EF1, which is distinct among solved structures of endolysins, consists of one full-length peptide associated with three additional N-terminally truncated CBDs fragments” (please see line 293-295). We are quite agreed with the referee that heterotetramer endolysins are widely spread. However, their structures are not determined.

Line 299-304. “…suggesting that the integrity of the structure can be maintained only in the presence of the three additional truncated CBDs.” Actually, there is no data that would support comments about structural integrity one way or another. The authors only show that the enzyme requires a multimeric assembly for maximal activity…this has nothing to do with structural integrity or stability.

Authors response: In this study, we find that when the interaction among the CBDs are disrupted, the lytic activity of LysIME-EF1 lost, suggesting that if the integrity of the holoenzyme is destroyed, it does not have activity. Now we described as “disruption of the oligomerization interface between CBDs results in significant decrease in lytic activity against *E. faecalis*, suggesting that LysIME-EF1 is a multimeric endolysin that requires the assembly of additional three CBDs to achieve an oligomeric conformation for optimal activity, a similar result was reported in other multimeric endolysin， such as the previous identified endolysin Lys170 from the *enterococcal* phage F170/08 or CTP1L, which lyses *Clostridium tyrobutyricum*” (please see line 297-302).

- Line 304-306. “We can further argue that, without the extra CBD fragments, the CBD of the full-length LysIME-EF1 would not be anchored in an optimal orientation to bind to the cell wall and carry out its function.” No, actually, you can’t say that. The authors have provided no evidence of enzyme kinetics, no evidence of binding constants/affinities between wild type and mutants, no co-crystals of LysIME-EF1 and cell wall epitopes/substrates to justify any such statements about anchoring to the bacterial surface or optimal orientation, even in a speculative discussion.

Authors response: Now we deleted it and described as “disruption of the oligomerization interface between CBDs results in significant decrease in lytic activity against *E. faecalis*, suggesting that LysIME-EF1 is a multimeric endolysin that requires the assembly of additional three CBDs to achieve an oligomeric conformation for optimal activity, a similar result was reported in other multimeric endolysin, such as the previous identified endolysin Lys170 from the *enterococcal* phage F170/08 or CTP1L, which lyses *Clostridium tyrobutyricum* [32,34]” (please see line 297-302).

- Line 311-314. The authors forgot about the work on PlyC….a multimeric endolysin not mentioned.

Authors response: We agreed with the referee that PlyC is a multimeric endolysin and we cited the related papers in our manuscript many times. However, in this sentence, we address that Lys170, CTP1L and CD27L, as well as LysIMEF1, are multimeric endolysins encoded by a single gene. Anyway, we much appreciate for the referee’s excellent review.

- In general, the methods section is very brief and not adequate. Some sections are entirely missing (i.e. analytical gel filtration, etc.)

Authors response: A more detail methods section is described in the revised version of the manuscript. We hope it may be more evident to the readers and it can satisfy the referee. We appreciate the referee’s excellent suggestion.
