# Supplementary material for: Structural and functional insights into a novel two-component endolysin encoded by a single gene in Enterococcus faecalis phage
Source: PLoS Pathog. 2020 Mar 16;16(3):e1008394. doi: 10.1371/journal.ppat.1008394 (PMC7098653; doi:10.1371/journal.ppat.1008394)
Supplement: Attachment — Submitted filename: Response to PPATHOGENS-D-19-01755R2-0203.docx [file ppat.1008394.s013.docx]

**Reviewer #1**

**Part I - Summary**
Please use this section to discuss strengths/weaknesses of study, novelty/significance, general execution and scholarship.

Reviewer #1: (No Response).

**Part II – Major Issues**: Key Experiments Required for Acceptance

Please use this section to detail the key new experiments or modifications of existing experiments that should be absolutely required to validate study conclusions.

Reviewer #1: (No Response).

**Part III – Minor Issues**: Editorial and Data Presentation Modifications

Please use this section for editorial suggestions as well as relatively minor modifications of existing data that would enhance clarity.

Reviewer #1: The revised manuscript is improved with additional experiments and has addressed most of my previous concerns. However, the authors chose to keep the results on animal model, which is all right to show some additional data but there are some things to be clarified.

1. Bacterial dose. In the materials and methods section, the authors evaluated the virulence of E. faecalis 02 with different bacterial dosage, i.e., 2×107, 2×108, 2×109 and 2×1010 CFU/mouse, to seek the minimal lethal dose and the authors said the minimal lethal dose of bacteria was used in the animal infection experiments (page 25, line 449). While, in the results section, bacterial dosage is ≥2 × 1010 cfu/mouse (page 15, line 272). And in the corresponding Figure 7 legend, the injected bacterial dosage is 4×109 cfu/mouse (page 30, line 519). These data are not consistent. What is the minimal lethal dose of the strain tested?

Response: We apologize for the ambiguity. The minimal lethal dose (MLD) of the *E. faecalis* 002 strain used in animal infection experiments was 2x10^9^ CFU. To determine the MLD, different doses of *E. faecalis* 002 were injected into mice, and the results indicated that 2x10^9^ CFU is sufficient to cause a 100% mortality rate within 4 days. After determining 2x10^9^ CFU as MLD, we decided to use 2xMLD (i.e. 4x10^9^ CFU) as the challenge dose. We revised the “Evaluation of protective capabilities” subsection of Results (lines 275-294 of the new manuscript) to ensure that this information is delivered more clearly to readers.

2. In the materials and methods section, survival rates of the animals were recorded every 8 h for the first 48 h and then every 12 h for up to 4 days post-infection. The survival rates shown in Figure 7 are the survival rates at 2 days post-infection? Why did the authors choose 4 days of observation and present the survival rates on day 2.

Response: We apologize for confusion caused by this oversight. In the first version of our manuscript, the data presented in Figure 7 were assessed by Kaplan–Meier survival analysis, but in the second version manuscript the original Figure 7 was modified to present only the calculated survival rates of mice. Prompted by the reviewer’s notice, we replace the current Figure 7 with the first version of Figure 7 that used Kaplan–Meier survival analysis, presents data more clearly, and matches textual description of the experiment and make the Figure 7 more clear.

3. In both methods section and figure legend, a number of n=8 animal/group was given. What do the error bars in Fig 7 stand for? Did the authors repeat the experiments (n=8 animal/group) for several times? If that is the case, the repeat times should be given accordingly. Also it looks odd for percentages of 96% mortality rate (Line 275), 95.83% (Line 276) and 16.66% (line 279) 54.17% (line 281), and 45.83% (line 282). How were these percentages calculated if the denominator is 8?

Response: We apologize for the confusion caused by the same reason as the issue raised in question 2. Replacement of the current Figure 7 with the original one that uses Kaplan–Meier survival analysis and matches the textual description of our experimental data analysis should clear up any confusion.

Minor points：

-Methods for Fig 1C and 1D, as well as Fig 5D etc were missing.

Response: The methods for Fig 1C, 1D and 5D are added to the Method section of the new manuscript (lines 428-432 and 443-446).

--Page 6, line 91, Fig S1 (p30, line 523), broader bactericidal spectrum. Please indicate which lysin LysIME-EF1 was compared to?

Response: LysIME-EF1 was found to lyse multiple strains of *E. faecalis* in our lab and the bactericidal activity was observed to be quite efficient, but we did not compare its bactericidal spectrum to any other lysin. Therefore, in the new manuscript (lines 105-107) we rephrase the sentence as follows: “Remarkably, LysIME-EF1 displays efficient bactericidal activity against 29 clinical strains of *E. faecalis* (S1A Fig).”

--page 15, lines 274-275, the sentence is confusing. Please change to “… variants (20 ug) 1 h post-infection. Mice that were not….”.

Response: The abovementioned sentence has been deleted because of rephrased sentences.

--page 20, site-directed mutagenesis. All primers used should be given in the supplementary.

Response: In the new version of manuscript we add the S1 Table which lists all the primers used in this study.

--Line 416, turbidity reduction assay. It’s better to use g instead of rpm? Please check other places.

Response: Following reviewer’s suggestion and to ensure the precise description of our experimental conditions, we express centrifugal conditions in units of relative centrifugal force (RCF, unit ×g) instead of revolutions per minute (rpm) for every centrifugation experiment in our study.

--page 24, line 424, OD600 should be shown as OD600.

Response: The abbreviation OD600 has been changed to OD_600_ on the page 24, line 436 of the original manuscript, as well as at every other mention.

--Line 428, PBS is not negative control, it is blank control.

Response: PBS was changed into blank control as pointed out by the reviewer (line 440 of the new manuscript).

--page 29, line 514 and line 516, delete the second LysIME-EF1.

Response: The second LysIME-EF1 has been deleted as the reviewer suggested.

--Page 30, line 519, what VREF stands for? A full name should be given when it was shown first time.

Response: As defined in line 93 of the new manuscript, VREF stands for vancomycin-resistant *E. facealis* 002. However, to avoid ambiguity, the abbreviation VREF was deleted from the manuscript except at the first mention of *E. facealis* 002. Therefore, in the new manuscript, this bacterial strain is referred to as *E. facealis* 002 throughout the manuscript.

--Page 30, line 523, delete r between broader and bactericidal.

Response: The r between broader and bactericidal has been deleted as the reviewer suggested.

**Reviewer #2**

**Part I - Summary**
Please use this section to discuss strengths/weaknesses of study, novelty/significance, general execution and scholarship.

Reviewer #2: The authors have done a good job at explaining some key points and carrying out additional informative experiments. I believe the paper is ready for publication.

**Part II – Major Issues**: Key Experiments Required for Acceptance

Please use this section to detail the key new experiments or modifications of existing experiments that should be absolutely required to validate study conclusions.

Reviewer #2: (No Response)

**Part III – Minor Issues**: Editorial and Data Presentation Modifications

Please use this section for editorial suggestions as well as relatively minor modifications of existing data that would enhance clarity.

Reviewer #2: (No Response)

**Reviewer #3**

**Part I - Summary**
Please use this section to discuss strengths/weaknesses of study, novelty/significance, general execution and scholarship.

Reviewer #3: I was reviewer 3 in the original manuscript and this revised manuscript is significantly improved. In particular, the authors have added new bacteriolytic assays for most experiments, added additional purification gels, shown standards on gel filtration, and included additional details for some of the methodology. Moreover, they have adequately addressed most (not all) reviewer comments to a reasonable level of satisfaction. Nonetheless, there still remain a couple of errors and questions that were not fully addressed, requiring some additional revisions.

**Part II – Major Issues:** Key Experiments Required for Acceptance

Please use this section to detail the key new experiments or modifications of existing experiments that should be absolutely required to validate study conclusions.

Reviewer #3: Line numbers in the comments below refer to the marked up version of the revised manuscript.

- Lines 108/109 and 122/123. The authors state that their enzyme “displays a broader host range than other homologous endolysins, with bactericidal activity against 29 clinical strains of E. faecalis”. What do they mean by this comment? A broader host range than what? The fact that they tested 29 strains does not make this broad activity or broad spectrum since all 29 strains were the exact same species. All endolysins show activity toward at least one species and many show activity toward multiple species. Thus, to make a statement about a broader host range than other endolysins when they only test one species is nonsensical.

Response: LysIME-EF1 was found to lyse multiple strains of *E. faecalis* in our lab and the bactericidal activity was observed to be quite efficient, but we did not compare its bactericidal spectrum to any other lysin. Therefore, in the new manuscript (lines 105-107) we rephrase the sentence as follows: “Remarkably, LysIME-EF1 displays efficient bactericidal activity against 29 clinical strains of *E. faecalis* (S1A Fig).”

- Line 209. The authors reference figure S3C, which does not exist. I believe they mean to reference figure S4C.

Response: The incorrect reference pointed out by the author was corrected.

- Line 225. The authors reference figures S4A and S4B, but I believe they mean to reference figures S5A and S5B.

Response: The incorrect references pointed out by the reviewer were corrected.

- Lines 313-325. Proenca et al. 2015 did this same bioinformatics analysis on Lys170 and found the same results (i.e. methionine as first amino acid residue of the CBD and upstream Shine-Dalgarno sequence suggesting an alternative start codon site that generates oligomer). At the least, the authors need to acknowledge this previous work. Perhaps a statement saying that their analysis is consistent with the findings of Proenca et. al or something along these lines.

Response: Thank you for pointing this out. The new version of the manuscripts states the following (lines 253-256): “These results are consistent with the previous bioinformatics analysis by Proenca et al [32] and imply that the use of a secondary translation initiation site may be widespread among endolysins targeting Enterococcus species”.

- Lines 332-336. The authors discuss residues 173, 176, and 190 and label these residues in figure 6A. This is fine; however, it would be very insightful to the reader if they also pointed out the location of these residues in Figure S9, which shows the electrostatic map of the planar surface so the reader can visualize exactly where these critical residues are located on the CBD surface.

Response: Following reviewer’s suggestion, we modified the Figure S9 so that the locations of the residues 173, 176 and 190 are pointed out and visible for reader’s convenience.

- Lines 338-340. The authors should insert a reference to Figure 6B at the end of this sentence describing the FACS results.

Response: A reference to Figure 6B has been inserted at the end of the sentence describing FACS results (lines 266 of the new manuscript).

- Line 346. The authors reference Figure 6B here, but I believe they mean to reference Figure S8 instead.

Response: Incorrect reference to Figure 6B at the line 274 of the old manuscript has been corrected to refer to Figure S8.

- Figure 7. This figure and all of the associated in vivo work is still fraught with issues. The authors have already shown that the mutants are deficient in antimicrobial activity, so to show the same thing in vivo is not very impactful. As another reviewer pointed out, the in vivo data provides no additional structural or mechanistic insight into LysIME-EF1, and I agree. I think the data should be removed. If the authors choose to keep the in vivo data, then many other issues will need to be addressed:

- In vivo, Issue 1. The authors did a pilot experiment to determine the minimum lethal dose (MLD) in lines 554-557. However, they never report what dose was experimentally determined to be the MLD. On line 353, it is stated that the mice were treated with 2 x 10^10 cfus, so one presumes this was the MLD, yet the figure legend (line 665) states the mice were treated with 4 x 10^9 cfus. Which is it? Also, the 4 x 10^9 dose was not one of the doses tested for MLD, so there is no rationale for how they derived this dose.

Response: We apologize for confusion caused by inconsistencies in our manuscript. In our pilot study, the minimal lethal dose (MLD) of the *E. faecalis* 002 strain was experimentally determined to be 2x10^9^ CFU. We therefore decided to use 2xMLD (i.e. 4x10^9^ CFU) as the challenge dose to treat the mice. The text was revised accordingly to present this information in a clearer way (lines 454-473 of the new manuscript).

- In vivo, Issue 2. It is stated that mice were divided into 10 groups of 8 mice (lines 559-560). Figure 7 only shows 8 groups (WT, control, and 6 mutants). What is the purpose of the 2 remaining groups?

Response: Thank you for pointing out this issue. As shown in Figure 7, *in vivo* animal experiments included 8 groups, not 10 as stated in lines 559-560 of the original manuscript. We revised the manuscript accordingly for correctness and consistency of information.

- In vivo, Issue 3. In both the original manuscript and the revised manuscript the authors consistently state that they used 8 mice for each group. However, in their rebuttal letter, they state they did the experiments in triplicate, indicating N=24 per group, not N=8 per group. This information is not stated anywhere in the manuscript. Were these new experiments since the original submission? If so, things need to be revised to state N=24 per group.

Response: The new manuscript and states n=24 per group (lines 286 of new manuscript).

- In vivo, Issue 4. Statistics. Error bars are presented, which is odd in an in vivo experiment where normally the total N value is reported. As stated above, the authors never signify that experiments were done in triplicate, but this must be the case as they report 95.83% protection for WT (line 357), which cannot be derived from a group of 8 mice, but can be derived from a group of 24 mice (i.e. 23 out of 24 protected). They should report the data this way (i.e. 23/24). More importantly, the authors present no statistical data to compare results between groups, which is a significant flaw and must be addressed.

Response: As mentioned above, the new manuscript signifies the total n value for each *in vivo* animal experiment groups. Moreover, we corrected our data presentation as suggested by the reviewer and it should be clearer in the new manuscript (lines 275-295 of the new manuscript).

- In vivo, Issue 5. What is VREF (lines 549 and 665). This term is never defined.

Response: As defined in line 93 of the new manuscript, VREF stands for vancomycin-resistant *E. facealis* 002. However, to avoid ambiguity, the abbreviation VREF was deleted from the manuscript except at the first mention of *E. facealis* 002. Therefore, in the new manuscript, this bacterial strain is referred to as *E. facealis* 002 throughout the manuscript.

- In vivo, Issue 6. The mice are monitored for 4 days according to the methods (lines 564-566). However, the authors state that the experiments were only 2 days in the results (line 356) and figure legend (line 667). Which is it?

Response: As stated in the Methods, the mice were indeed monitored for 4 days. However, the fact that mortality rates of the mice did not change from day 2 to day 4 was important when we decided to present data in a bar graph. As suggested by the reviewer in Issue 9, we replaced the current bar graph with the Kaplan-Meier survival curves from the original manuscript, which overall helps with clarity in data presentation.

- In vivo, Issue 7. Lines 354-356 are awkward. I think some sentences were combined or there is an extra period in line 355.

Response: The text has been revised for improved readability.

- In vivo, Issue 8. The results only provide life or death metrics. Was there any attempt to measure health status…i.e. even if a mouse is alive at day 2 or day 4 does not mean the mouse is healthy or otherwise will not die the next day, which is why these types of experiments should either last longer than a few days or should include some type of health monitoring matrix.

Response: Thank you for your kind suggestion. The infection model used in our study is based on methods described previously (the reference 35 in the new manuscript), and unfortunately we did not attempt to measure health status but paid attention to mortality rates only. As shown in the Figure 7 of the new manuscript (same as Figure 7 of the original manuscript as suggested by the reviewer), the data obtained from *in vivo* animal experiments were assessed by Kaplan–Meier survival analysis.

- In vivo, Issue 9. Graph. In the original manuscript, the authors presented the data in Kaplan Meier survival curves, which was appropriate. However, because several lines overlapped, they chose to present the data in a bar graph in the revised manuscript. This is not advisable because it loses all the temporal associated data displayed in a Kaplan Meier graph. I would go back to the KM graph even if multiple KM graphs are needed due to the overlap.

Response: Following reviewer’s suggestion, we replaced the current Figure 7 with the original Figure 7 that presents data in Kaplan-Meier survival curves.

- Final conclusions. In lines 422-423 the authors state that residue R190 is essential for binding to the host cell wall and hence the biological activity of the endolysin. They then state in lines 424-26 that alteration of this residue could provide an opportunity to design endolysins with high specificity, efficacy, or broader spectrum. If R190 is essential for binding, it is not clear how alteration of this residue in particular can potentiate all of these actions. While I agree that the authors have described essential residues and mechanisms that increase our general understanding of endolysins and this general understanding may one day inform future engineering efforts, I think the comments specifically aimed at alteration of residue R190 without supporting data is a bit overstated.

Response: We agree that the comments aimed at alteration of residue R190 are overstated and apologize for this mistake. We revised our conclusion, which now states the following (lines 331-334 of the new manuscript): “Based on the results of the structural and biochemical analysis, it was demonstrated that the residue R190 is essential for LysIME-EF1 binding to host cell. The animal experiments performed in this study further corroborated the results of structural and biochemical analysis.”

- Turbidity reduction assays. In lines 521-528 the authors describe the general methods for the turbidity reduction assay. In the revised manuscript, all of the turbidity reduction assay experiments have been removed and replaced by the bacteriolytic assays. The one exception is the experiment with EDTA and metal ions presented in Figure 3D. Therefore, rather than general methods, these methods should specifically include experimental details for the EDTA/metal ion data. For example, specify how much EDTA was used, describe the dialysis steps, and describe the supplemental addition of metal ions.

Response: The authors thank the reviewer for the suggestion. The new manuscript includes specific experimental details regarding the EDTA/metal ion data, including the concentration of EDTA used, dialysis step and concentration of metal ions added to the EDTA-inactivated LysIME-EF1.

**Part III – Minor Issues**: Editorial and Data Presentation Modifications

Please use this section for editorial suggestions as well as relatively minor modifications of existing data that would enhance clarity.

Reviewer #3: (No Response).
